# Droplet Microfluidics: From Generation to Manipulation

**DOI:** 10.3390/mi16121359

**Published:** 2025-11-29

**Authors:** Nan Xiang, Lin Jiang, Zhonghua Ni

**Affiliations:** School of Mechanical Engineering, and Jiangsu Key Laboratory for Design and Manufacturing of Precision Medicine Equipment, Southeast University, Nanjing 211189, China; lin-jiang@seu.edu.cn

Microfluidics is a powerful technique that manipulates fluid flow within microscale channels, enabling highly precise and reproducible fluid control in extremely small confined spaces [1,2,3]. This capacity allows for exceptional efficiency and sensitivity when analyzing and processing minute-volume samples. By leveraging its unique advantages, microfluidics facilitates various novel functions such as sample preparation, biochemical reactions, material synthesis, and single-cell analysis, and thus plays a significant role in biological engineering, chemical engineering, clinical diagnosis, and even electronic engineering [4,5,6].

As an important branch of microfluidic technology, droplet microfluidics commonly utilizes hydrodynamic effects to segment the discrete phase within immiscible fluids, generating femtoliter- to nanoliter-volume microdroplets [7,8]. There are two main types of droplet generation strategy [9,10]. The first type relies on the shear force to segment the fluid and includes the cross-flow, flow-focusing, and co-flow, while the second includes the step-emulsion geometry, which employs the interfacial tension to generate droplets. The throughput of droplet generation can reach a frequency in the order of up to kHz. Through the engineering of more immiscible liquids using a multiple-capillary device, the generation of droplets with diverse morphologies or complex interior structures can be realized, providing new insights for engineered particle preparation [11,12].

After the controllable generation of microdroplets, droplet microfluidics can realize the mixing, splitting, moving, merging, and sorting of droplets within microchannels or microchambers, enabling effective sample processing, such as on-chip incubation, reagent addition, real-time tracking, and sample cleanup [13]. By simultaneously processing thousands of tiny droplets, droplet microfluidics allows for the rapid screening and analysis of vast quantities of biological samples, significantly enhancing experimental throughput and efficiency. In addition, droplet microfluidics provides highly isolated environments at the microscale, greatly reducing the risk of cross-contamination between samples. The internal conditions within the droplets, such as temperature, pH, ionic concentration, and other biochemical parameters, can be precisely controlled. It is concluded that the main advantages of droplet microfluidics include uniform and controllable droplet size, low reagent consumption, high throughput, high efficiency, excellent experimental reproducibility, minimal cross-contamination, and ease of precise manipulation. Up until now, droplet microfluidics has been widely applied in single-cell analysis, protein engineering, enzyme activity assays, tissue engineering, and synthetic biology [14]. For example, researchers can utilize microdroplets to encapsulate and analyze individual cells, providing new tools for understanding cellular behavior and complex disease mechanisms [15]. In addition, as the droplets remain stable for a long time without coalescence, the droplet microfluidics can be used as the reaction and detection microchambers of polymerase chain reactions (PCRs), loop-mediated isothermal amplification (LAMP), and enzymatic amplification [15,16,17]. The third-generation digital droplet PCR (ddPCR) technology is derived from the digital capabilities of droplet microfluidics and enables the absolute quantification of target nucleic acid molecules [18]. The core strategy of ddPCR involves partitioning nucleic acid molecules into individual droplets, followed by independent amplification within each droplet until detectable signals are achieved.

In recent years, the integration of droplet microfluidics with newly emerging technologies (multispectral detection, high-speed imaging, and artificial intelligence) has driven the rapid development of droplet microfluidics [19,20]. By integrating high-speed imaging, multispectral detection, and intelligent algorithms, the flowing droplets can be monitored and analyzed in real time, which enables efficient automated droplet detection and on-demand sorting based on complex characteristics (e.g., size, shape, biomarker, or chemical composition) of targets encapsulated in droplets [21,22]. In algae breeding, droplet microfluidics can rapidly and accurately screen and sort algal cells, optimizing production processes for biofuels and other biological products [23,24]. It can effectively identify and select algal cells with high productivity or specific biochemical characteristics, promoting the sustainable development of renewable energy and biological resources. In liquid biopsy, droplet microfluidics has recently become a highly sensitive single-cell analysis tool for the quantitative detection of the heterogeneity of rare circulating tumor cells in blood, aiding in the early diagnosis and prognosis assessment of cancer [25,26].

Our Special Issue, “Recent Advances in Droplet Microfluidics”, is devoted to highlighting the most recent advances in droplet microfluidics. Four outstanding papers are included in this Special Issue. We will now briefly introduce these papers.

Three of the papers in this Special Issue concern droplet production technology. Traditional droplet generation technologies commonly require complex and expensive experimental setups (such as pumps). To address this issue, Yetiskin et al. [27] developed a low-cost and pump-free droplet generator using off-the-shelf products, including a driver board, a piezo-ring transducer, a metal sheet with holes, and a 3D-printed part assembly. Their device could generate oil-in-water droplets with average diameters of 4.62 ± 0.67 μm without using external pumps, providing a simple, cost-effective, and scalable tool for large-scale generation of droplets with uniform sizes.

Step emulsification is a robust method for generating monodisperse emulsion droplets using arrayed nozzles. However, the production of monodisperse oil-in-water droplets and polymeric microspheres below 20 µm in diameter using poly(dimethylsiloxane) (PDMS)-based step emulsification devices is still rarely reported. Tottori et al. [28] developed a scalable PDMS-based step emulsification device for the continuous production of monodisperse oil-in-water droplets and polymeric microspheres below 20 µm in diameter. By comparing straight and triangular nozzle designs, they demonstrated that the triangular configuration consistently generated droplets with diameters under 20 µm and coefficients of variation below 4%, achieving a maximum throughput of 0.5 mL·h^−1^. These droplets were successfully photopolymerized off-chip into uniform acrylic microspheres. The disposable device offers a scalable and cost-effective route to create precise microparticles suitable for both lab and industrial applications.

Microfluidic granulation offers a promising approach for the high-quality production of energy-dense microspheres. Nevertheless, its wider adoption remains constrained by several limitations, including a narrow operating range, a low production yield, and a lack of process continuity. To address these limitations, Liu et al. [29] developed a microfluidic granulation system using printing, co-flow, and flow-focusing. Their system achieves the continuous, high-speed generation of microspheres with tunable diameters ranging from 110 to 2500 µm, a coefficient of variation as low as 1.9%, operating frequencies over 13 kHz, and a suspension consumption rate of 100 mL/h. Using sodium alginate hydrogel as a binder, they fabricated calcium alginate/perchlorate composite microspheres with a uniform morphology, narrow size distribution, and high active material loading.

Instead of droplet generation and microsphere production, Parsi et al. [30] studied the mechanism of droplet actuation using electrowetting on dielectric (EWOD). EWOD is a technique that controls the wetting behavior of a liquid droplet on a solid surface by applying an external electric field. They developed an analytical model to explore performance trade-offs for actuating a panel using EWOD forces in RF devices. The effectiveness of the developed model was validated by experiments, achieving less than 7.8% error in predicting plate velocity under varying voltages. A 3D FEM model was further established to analyze velocity profiles and viscous forces across the droplet height, offering insights beyond experimental capabilities.

It is anticipated that droplet microfluidics will exert transformative effects on biochemical research and clinical diagnostics.
